# Analyzing Bunyaviruses from a One Health Perspective

**DOI:** 10.3390/v18101110

**Published:** 2026-10-08

**Authors:** Daniel Desmecht, Hani Boshra

**Affiliations:** Department of Morphology and Pathology, Fundamental and Applied Research for Animals & Health (FARAH), Faculty of Veterinary Medicine, University of Liège, 4000 Liege, Belgium; daniel.desmecht@uliege.be

**Keywords:** One Health, bunyaviruses, Rift Valley fever virus, Crimean Congo hemorrhagic fever virus, Hantaviruses

## Abstract

Zoonotic viral diseases continue to pose major challenges to global health, particularly as environmental, social and economic changes increasingly influence interactions among humans, animals, vectors, and ecosystems. This review explores Rift Valley fever virus (RVFV), Crimean-Congo hemorrhagic fever virus (CCHFV), and hantaviruses (HTVs) within the framework of One Health, emphasizing the ecological and epidemiological factors that govern their emergence and transmission. Despite their distinct transmission cycles, these viruses share important determinants of zoonotic risk. RVFV is primarily maintained through interactions between mosquitoes, livestock, wildlife, and humans; CCHFV is sustained within complex tick–vertebrate systems involving *Hyalomma* ticks, domestic animals, and wildlife, while HTVs are predominantly maintained through persistent infections in mammalian reservoir hosts, particularly rodents. Climate variability, habitat alteration, agricultural expansion, livestock movement, urbanization, and other anthropogenic pressures can modify these transmission systems and create new opportunities for pathogen spillover. Effective prevention and control therefore require integrated surveillance that encompasses human disease, animal populations, vectors or wildlife reservoirs, and environmental conditions. Advances in molecular diagnostics, genomic epidemiology, ecological modeling, remote sensing, and climate-based forecasting provide valuable opportunities for improving early detection and outbreak preparedness. To reduce the burden of these zoonotic viral diseases, a sustained collaboration among human and veterinary health professionals, ecologists, entomologists, environmental scientists, and public health authorities is required. Therefore, a coordinated One Health approach is essential for anticipating emerging threats, strengthening preparedness, and protecting human, animal, and ecosystem health.

## 1. The One Health Framework, Its History and Evolution

**Figure 1:**
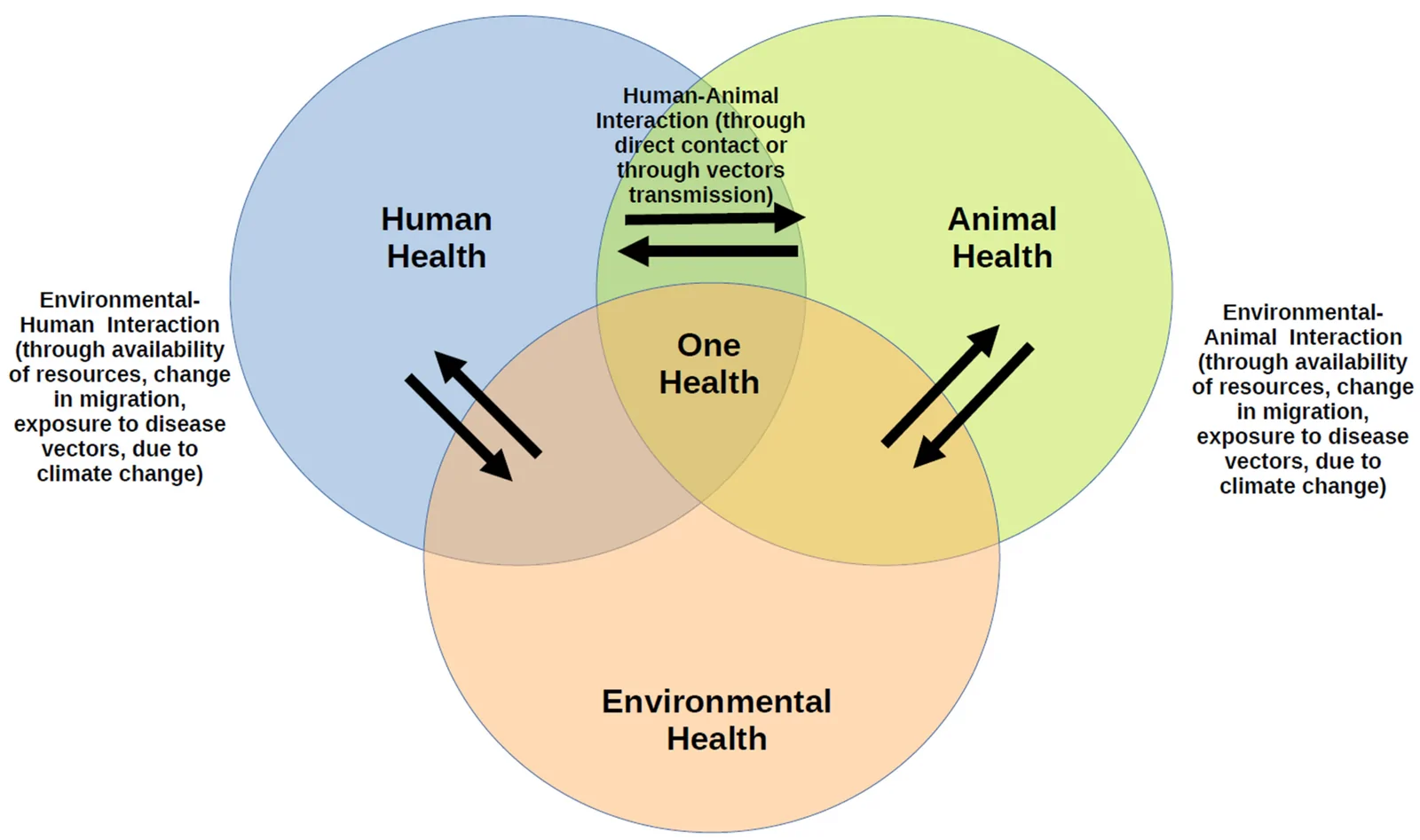
Schematic describing the principle of One Health. This concept is based on the interaction of three elements: Human, Animal and Environmental Health. The interaction between Human and Animal Health plays a significant role in the transmission of zoonotic diseases. Furthermore, the role of Environmental Health, with regard to climate change and man-made changes to habitat, also affects both Human and Animal Health.

The One Health framework is an interdisciplinary approach that recognizes the intrinsic connections between human health, animal health, and environmental health (Figure 1). Although the concept that veterinary medicine is analogous to human medicine can be traced back to ancient Egypt [1,2], the origins of the One Health concept can be traced to the late eighteenth and nineteenth centuries through the work of comparative anatomists and physicians who recognized similarities between human and animal diseases [3,4].

One of the earliest and most influential proponents was Rudolf Virchow (1821–1902), the German physician and pathologist widely regarded as the father of modern pathology. His famous statement, “Between animal and human medicine there are no dividing lines—nor should there be,” has become one of the philosophical cornerstones of the modern One Health movement [5,6].

Virchow’s ideas strongly influenced his student Sir William Osler (1849–1919), who consistently advocated for integrating veterinary and human medicine in medical education and research, emphasizing that understanding animal diseases could greatly improve knowledge of human pathology [5,6]. Together, the work of Virchow and Osler established the scientific foundation for comparative medicine, which later evolved into the concept of One Medicine.

The twentieth century witnessed the formalization of these ideas through the work of Calvin W. Schwabe, an American veterinary epidemiologist widely regarded as one of the fathers of modern One Health. In his influential textbook *Veterinary Medicine and Human Health*, first published in 1964, Schwabe introduced the concept of “One Medicine,” arguing that human and veterinary medicine represent complementary disciplines grounded in the same biological principles [7]. His work laid the intellectual foundation upon which the modern One Health framework was subsequently built [4].

Despite these conceptual advances, human medicine, veterinary medicine, and environmental sciences remained largely separated throughout much of the twentieth century because of increasing specialization within biomedical research and healthcare systems [8].

By the late twentieth century, scientists increasingly recognized that most newly emerging infectious diseases originated in animals. Taylor et al. [9] estimated that approximately 60% of known human infectious diseases are zoonotic, while nearly 75% of emerging infectious diseases originate from wildlife or domestic animals. Jones et al. [8] further demonstrated that the frequency of emerging infectious diseases had increased substantially since the 1940s, with wildlife serving as the primary reservoir for many novel pathogens.

Major disease outbreaks—including HIV/AIDS, Nipah virus, Hendra virus, West Nile virus, SARS, highly pathogenic avian influenza (H5N1), and Middle East Respiratory Syndrome (MERS)—provided compelling evidence that ecological disruption, wildlife trade, globalization, agricultural intensification, urbanization, and environmental degradation collectively influence pathogen emergence [4,8].

A major turning point occurred in 2004 with the publication of the *Manhattan Principles* during a symposium organized by the Wildlife Conservation Society [10]. Unlike Schwabe’s concept of One Medicine, which primarily linked veterinary and human medicine, the Manhattan Principles explicitly incorporated environmental conservation as a central determinant of health, thereby establishing the conceptual framework for modern One Health [3,10].

Following the Manhattan Principles, international organizations rapidly embraced the One Health approach, which now includes the World Health Organization (WHO), the Food and Agriculture Organization of the United Nations (FAO), the World Organisation for Animal Health (WOAH, formerly OIE), and more recently, the United Nations Environment Programme (UNEP) [11].

The COVID-19 pandemic represented perhaps the strongest demonstration of the importance of the One Health framework. Although investigations into the origins of SARS-CoV-2 continue, the pandemic highlighted the enormous societal and economic consequences of zoonotic spillover events and reinforced the need for integrated surveillance systems that monitor pathogens circulating among wildlife, domestic animals, and human populations simultaneously [11].

Today, One Health extends well beyond emerging infectious diseases. Factors such as climate change influence the distribution of disease vectors, alter wildlife migration patterns, and increase the risk of emerging infectious diseases through changing ecological interactions [3]. Advances in genomics, artificial intelligence, environmental surveillance, and molecular epidemiology increasingly facilitate integrated approaches to disease monitoring and prevention [11]. As globalization, urbanization, biodiversity loss, and climate change continue to reshape disease ecology, One Health is expected to play an increasingly central role in pandemic preparedness and sustainable public health policy [3].

While a plethora of diseases can be viewed through the One Health approach, this narrative review will focus on three members of the class *Bunyaviricetes* that threaten both human and veterinary health [12,13]: Rift Valley fever virus (RVFV), Crimean Congo hemorrhagic fever virus (CCHFV) and hantaviruses HTVs). The Authors conducted a literature search primarily through PubMed, using the search terms associated with each pathogen (i.e., Rift Valley fever, Crimean Congo hemorrhagic fever, hantavirus) along with the term “One Health”. While references were primarily focused on publications from 2000 onwards, older papers were also referenced if they served as the source material for a particular publication or were of a historical relevance (i.e., the history of One Health). The geographic distribution of each pathogen was determined using ProMedMail (www.promedmail.org) provided by the International Society for Infectious Diseases.

## 2. Importance of the One Health Approach with Regard to Bunyaviruses

Discussing the One Health approach is particularly important for understanding and addressing the public health risks posed by RVFV, CCHFV, and HTVs because the epidemiology of these pathogens extends beyond the human host and is fundamentally shaped by interactions among animals, vectors or wildlife reservoirs, and the environment. Although these viruses differ in their transmission mechanisms, all three are affected by environmental conditions, climate variability, land-use change, and human activities. Consequently, human disease surveillance alone is insufficient to identify and control emerging risks. A One Health framework enables information from veterinary medicine, wildlife ecology, entomology, environmental sciences, epidemiology, and clinical medicine to be integrated into coordinated surveillance and prevention strategies. Such an approach can facilitate earlier detection of pathogen circulation in animal or vector populations, identify environmental conditions associated with increased spillover risk, and support targeted interventions before human outbreaks become widespread. Examining these three viruses collectively therefore demonstrates how One Health can provide a practical and adaptable framework for anticipating zoonotic disease emergence, strengthening public health preparedness, and reducing the impact of environmentally driven infectious disease threats.

### 2.1. Rift Valley Fever Virus: A One Health Perspective

Rift Valley fever virus (RVFV) is a mosquito-borne zoonotic pathogen of major public health and veterinary importance, particularly in Africa and the Arabian Peninsula. The virus is widely regarded as a quintessential example of the One Health concept because its maintenance and transmission rely on the close interactions among humans, domestic animals, wildlife, arthropod vectors, and the environment [11,14,15]. Unlike many zoonotic pathogens that are maintained primarily within wildlife reservoirs, RVFV circulates through a complex ecological system involving mosquito vectors, livestock hosts, wildlife populations, climatic conditions, and human activities. Consequently, understanding the epidemiology and control of RVF requires collaboration across multiple disciplines, including veterinary and human medicine, entomology, ecology, climatology, molecular epidemiology, and environmental science.

**Figure 2:**
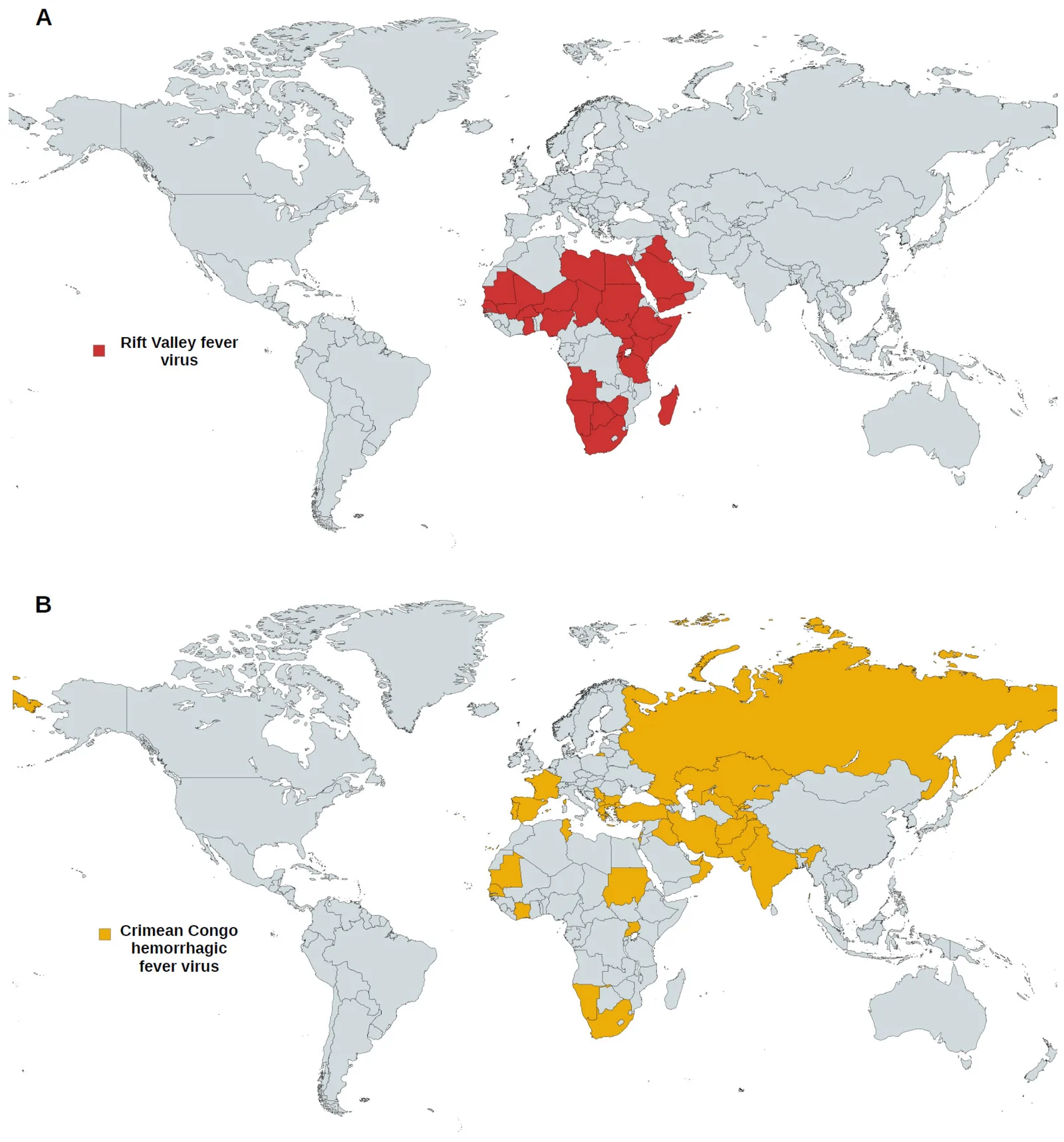

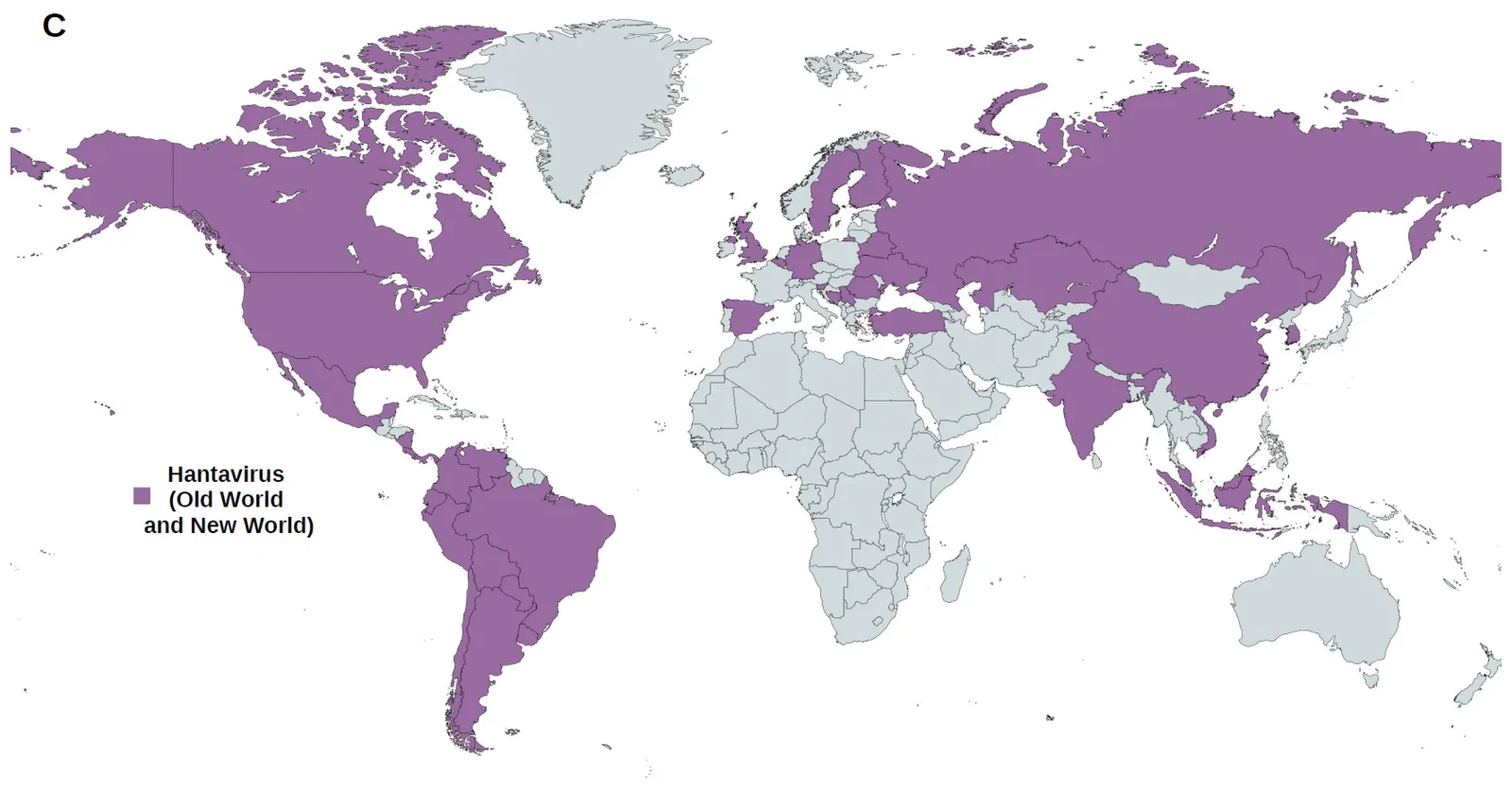
Geographic distribution of RVFV ((**A**)-red), CCHFV ((**B**)-yellow) and HTV ((**C**)-purple). The countries highlighted are based on reports of human, animal, or vector presence that were reported to public health officials and made available using ProMedMail (www.promedmail.org), provided by the International Society for Infectious Diseases.

RVFV is classified within the genus *Phlebovirus* of the family *Phenuiviridae* and contains a tripartite, negative-sense RNA genome composed of the L, M, and S genome segments [16]. The virus was first identified in 1930 during investigations into outbreaks of abortion and high mortality among sheep in Kenya’s Rift Valley by Daubney, Hudson, and Garnham [17]. Since its initial discovery, RVFV has become established throughout much of sub-Saharan Africa, where it causes periodic epizootics and epidemics affecting both livestock and humans. Its emergence outside Africa during the 2000–2001 outbreaks in Saudi Arabia and Yemen demonstrated the virus’s ability to spread beyond its traditional range through livestock movement and the presence of competent mosquito vectors, raising concerns regarding its potential for further international expansion [18,19]. Its current geographic distribution is shown in Figure 2A.

The appearance of RVFV beyond the African continent fundamentally altered its status from a regionally important veterinary pathogen to a disease of global concern. Increasing international trade, transboundary livestock movement, environmental change, globalization, and climate variability have created favorable conditions for the introduction of the virus into regions that support competent mosquito vectors, including parts of southern Europe and western Asia [20,21]. Reflecting this growing concern, the World Health Organization has designated RVFV as a priority pathogen because of its epidemic potential and the current lack of an approved vaccine for human use [22].

The transmission cycle of RVFV illustrates the central principles of the One Health framework. Rather than being maintained within a single host species, the virus circulates through an interconnected network involving mosquitoes, domestic ruminants, wildlife, and humans. Mosquitoes function not only as vectors but also as long-term reservoirs capable of maintaining viral circulation, whereas domestic livestock act as efficient amplification hosts by developing high levels of viremia that facilitate infection of feeding mosquitoes [15,23]. Human infection occurs through two principal routes: bites from infected mosquitoes and direct contact with infectious blood, tissues, or body fluids during activities such as animal husbandry, veterinary practice, slaughtering, or the handling of aborted fetuses [14,24].

Domestic ruminants represent the principal amplifying hosts responsible for sustaining RVFV transmission during outbreaks. Sheep are particularly susceptible, with infections frequently resulting in severe hepatic disease, high viral titers, extensive abortion, and mortality rates approaching 90–100% among newborn lambs, mainly of European breeds [14,17]. Pregnant ewes commonly experience abortion rates exceeding 80%, producing the characteristic “abortion storms” that often provide the first indication of RVF activity within affected regions [23]. Although disease severity is generally lower in cattle, goats, buffalo, and camels, these species nevertheless develop sufficient viremia to infect mosquito vectors efficiently, thereby contributing significantly to viral amplification and maintenance within endemic ecosystems [15].

Wildlife species also play an important role in the ecology of RVFV, although the extent of their contribution to long-term viral maintenance remains incompletely understood. Serological evidence of infection has been documented in numerous wild mammals, including African buffaloes (*Syncerus caffer*), antelopes, elephants, giraffes, rhinoceroses, and other free-ranging ruminants [25,26]. The close interaction between wildlife and domestic livestock in pastoral landscapes, protected areas, and mixed agricultural ecosystems creates opportunities for virus transmission among wildlife, livestock, mosquitoes, and humans. Consequently, surveillance of wildlife populations has become an increasingly valuable component of integrated One Health surveillance programs aimed at improving early detection of RVFV circulation [27].

Patterns of livestock production and animal movement exert a profound influence on the epidemiology of RVFV. Factors such as intensive farming practices, communal grazing, seasonal transhumance, unrestricted livestock movement, and transboundary animal trade facilitate the dissemination of infected animals into susceptible regions, where they may introduce the virus into local mosquito populations and initiate new transmission cycles [20,28]. For this reason, veterinary surveillance, regulation of animal movement, and strategic livestock vaccination remain among the most effective One Health interventions for simultaneously safeguarding animal health, reducing human infections, and protecting agricultural economies [22].

The consequences of RVF extend beyond disease in animals and humans, producing substantial economic and social impacts. Major outbreaks are associated with extensive livestock mortality, widespread abortion, reductions in meat and milk production, and restrictions on domestic and international livestock trade, resulting in considerable financial losses for affected countries [27,29]. In many endemic regions, livestock represent a family’s principal source of income, food security, transportation, and social status. Consequently, RVF disproportionately affects rural communities that frequently have limited access to veterinary and healthcare services. These broad societal impacts highlight that the One Health concept encompasses economic resilience and community well-being in addition to biological interactions among hosts, pathogens, and the environment.

Human disease occurs predominantly among individuals whose occupations involve close contact with livestock. Farmers, pastoralists, veterinarians, slaughterhouse workers, butchers, dairy workers, laboratory personnel, and other animal health professionals face an elevated risk of infection because of their routine exposure to infected blood, aborted fetuses, placental tissues, and animal carcasses [22,24]. Epidemiological studies have consistently identified slaughtering infected animals, assisting with animal births, handling aborted materials, and consuming raw animal products as important risk factors during outbreaks [30,31]. In contrast, mosquito-borne transmission predominates among residents of endemic regions during periods of excessive rainfall and flooding that promote explosive mosquito population growth.

Clinical disease in humans is usually mild, with most infections either remaining asymptomatic or presenting as an acute febrile illness characterized by fever, headache, muscle pain, joint pain, and generalized malaise [22]. Nevertheless, approximately 1–2% of infected individuals develop severe complications, including hepatitis, retinitis, meningoencephalitis, hemorrhagic disease, disseminated intravascular coagulation, and multiorgan failure [16]. Ocular involvement represents one of the most serious long-term complications because retinal lesions may result in permanent visual impairment or blindness. Although the overall case-fatality rate is relatively low compared with several other viral hemorrhagic fevers, mortality among patients with severe hemorrhagic disease may exceed 40–50% [23].

One of the defining epidemiological features of RVF is that outbreaks among livestock typically precede the appearance of human cases. This predictable sequence provides a valuable opportunity for early intervention because veterinary surveillance can serve as an effective warning system for impending human epidemics. Monitoring livestock abortion rates, conducting serological surveys, performing molecular diagnostic testing, and integrating mosquito surveillance frequently allow viral activity to be detected weeks before widespread human transmission occurs [32,33]. Close collaboration between veterinary and public health authorities therefore plays a critical role in strengthening outbreak preparedness and facilitating timely implementation of control measures.

Accordingly, the management of RVF has evolved from traditional disease control toward comprehensive One Health surveillance systems that integrate veterinary medicine, public health, wildlife ecology, entomology, climatology, and environmental monitoring. This multidisciplinary approach enables earlier detection of viral circulation, supports targeted livestock vaccination campaigns, guides vector-control strategies, informs occupational health interventions, and improves public health preparedness before large-scale outbreaks develop [22,27]. As a result, RVF has become one of the most compelling examples of how the One Health framework can be translated into effective surveillance and disease prevention strategies for emerging zoonotic pathogens.

Environmental conditions play a pivotal role in the epidemiology of RVFV, making the disease one of the most compelling examples of the intricate relationship between ecological processes and zoonotic disease emergence. Unlike many mosquito-borne pathogens that exhibit relatively predictable seasonal transmission, RVFV outbreaks are closely linked to episodic environmental events, particularly periods of excessive rainfall and flooding. These climatic conditions promote the formation of temporary wetlands and floodwater habitats that support explosive mosquito breeding, thereby increasing the likelihood of viral transmission among livestock and humans [15,34,35]. Consequently, environmental surveillance has become an essential component of RVF prevention and exemplifies the environmental pillar of the One Health framework.

Mosquitoes are indispensable to the maintenance and spread of RVFV because they function as both biological vectors and reservoirs of infection. Floodwater mosquitoes belonging primarily to the genus *Aedes* are considered the principal maintenance vectors, largely because infected females can transmit the virus vertically to their offspring through infected eggs [36]. This mechanism enables RVFV to persist during prolonged interepidemic periods even when susceptible vertebrate hosts are scarce. Following heavy rainfall, dormant eggs hatch simultaneously, producing infected adult mosquitoes capable of initiating transmission to nearby livestock populations [15]. As amplification of the virus occurs in domestic animals, numerous secondary mosquito vectors—including species of *Culex*, *Anopheles*, and *Mansonia*—become infected and contribute to rapid geographic dissemination of the virus [23,36]. The participation of multiple vector species substantially complicates mosquito control efforts and contributes to the rapid expansion of outbreaks.

Climatic variability is recognized as one of the strongest environmental drivers influencing RVFV emergence. In eastern and southern Africa, outbreaks are strongly associated with El Niño–Southern Oscillation (ENSO) events that generate unusually heavy rainfall and extensive flooding, creating ideal breeding conditions for mosquito populations [35,37]. Comparable relationships have also been documented in western Africa, Madagascar, and the Arabian Peninsula, where increased precipitation has consistently preceded major RVF epidemics [20]. Climate change is expected to further influence these transmission dynamics by increasing the frequency of extreme weather events, altering the geographic distribution of competent mosquito vectors, and extending seasonal periods favorable for viral transmission [38]. These observations underscore the importance of incorporating climate science into One Health surveillance and preparedness programs.

The development of remote sensing technologies has transformed RVF surveillance by enabling prediction of outbreaks before widespread viral transmission occurs. Satellite-derived measurements of rainfall, vegetation density, and surface water accumulation provide reliable indicators of ecological conditions that favor mosquito proliferation [34,35]. Monitoring parameters such as the Normalized Difference Vegetation Index (NDVI), rainfall anomalies, and sea surface temperature patterns allows public health agencies to anticipate periods of increased RVF risk several weeks or even months in advance. These forecasting systems support proactive interventions including targeted livestock vaccination, enhanced mosquito surveillance, movement restrictions on livestock, and public health preparedness. RVF therefore represents one of the earliest and most successful examples of integrating environmental monitoring technologies into infectious disease forecasting within a One Health framework.

Effective control of RVFV depends upon comprehensive surveillance systems that integrate animal, human, vector, and environmental health data. Veterinary surveillance often provides the earliest indication of viral activity through reports of abortion storms, increased neonatal mortality, and laboratory-confirmed infections in livestock [32]. Concurrent entomological surveillance enables monitoring of mosquito abundance, species composition, and viral infection rates, while environmental surveillance identifies ecological conditions associated with increased vector populations [33]. Human surveillance complements these efforts by facilitating rapid clinical recognition, laboratory confirmation, epidemiological investigation, and public health communication. Collectively, these integrated surveillance strategies provide a more complete understanding of RVFV transmission than any individual discipline could achieve independently and illustrate the practical implementation of One Health principles [27].

Recent advances in molecular epidemiology have further enhanced the capacity to monitor RVFV transmission. Whole-genome sequencing and phylogenetic analyses permit detailed characterization of viral evolution, movement across geographic regions, and patterns of introduction during outbreaks [16,39]. Genomic investigations conducted during recent epidemics in countries including Kenya, Mauritania, Senegal, and Rwanda have demonstrated repeated transboundary movement of RVFV associated with livestock trade and vector dispersal [22,40]. When integrated with epidemiological, veterinary, and environmental data, genomic surveillance provides valuable insight into transmission pathways and strengthens regional preparedness for future outbreaks.

Vaccination of livestock remains the cornerstone of RVF prevention because reducing infection in animal populations simultaneously decreases the risk of human disease. Several veterinary vaccines, including the live-attenuated Smithburn vaccine, Clone 13 vaccine, and inactivated vaccines, are currently available for use in endemic regions [16,23]. Although each vaccine differs with respect to efficacy, duration of immunity, and safety in pregnant animals, strategic vaccination programs have consistently reduced livestock mortality and limited zoonotic transmission during outbreaks [27]. In contrast, no widely licensed vaccine is currently available for human use, emphasizing the continued importance of livestock immunization, vector control, and occupational protection as the primary preventive measures [22].

Recognition of RVF as a transboundary zoonosis has reinforced the need for international collaboration. Organizations including the World Health Organization (WHO), the Food and Agriculture Organization of the United Nations (FAO), the World Organisation for Animal Health (WOAH), and the United Nations Environment Programme (UNEP) advocate coordinated surveillance and response strategies that integrate veterinary medicine, environmental science, entomology, and public health [11]. The Joint One Health Plan of Action identifies RVF as a priority example of a disease requiring multidisciplinary cooperation and emphasizes the importance of coordinated surveillance, laboratory capacity, information sharing, and outbreak preparedness. Increasingly, endemic countries have established national One Health platforms that facilitate communication among medical, veterinary, environmental, and agricultural sectors during outbreak investigations and response activities.

Experiences from recent epidemics have further demonstrated the effectiveness of integrated One Health interventions. Outbreak responses in Mauritania, Senegal, Kenya, Madagascar, Rwanda, South Africa, and the Comoros have combined livestock vaccination campaigns, vector-control measures, restrictions on animal movement, wildlife monitoring, enhanced laboratory diagnostics, human disease surveillance, and community education to reduce viral transmission [22]. These multidisciplinary responses have shown that successful control of RVF requires coordinated action across multiple sectors rather than reliance on medical or veterinary interventions alone.

Despite considerable progress, important challenges remain in the global control of RVFV. Many endemic countries continue to face limitations in surveillance infrastructure, diagnostic capacity, laboratory resources, and access to veterinary vaccines, particularly in remote rural areas [32]. Moreover, expanding irrigation systems, increasing livestock trade, land-use changes, and ongoing climate change are expected to alter mosquito ecology and facilitate viral emergence in new geographic regions. Addressing these challenges will require sustained investment in integrated surveillance systems, improved diagnostic capabilities, ecological research, predictive modeling, and strengthened collaboration among public health, veterinary, agricultural, and environmental agencies.

In summary, Rift Valley fever remains one of the most illustrative examples of a zoonotic disease that can only be effectively understood and controlled through a One Health approach. The complex interactions among mosquito vectors, domestic animals, wildlife, humans, and environmental factors underpin every stage of the viral transmission cycle. Continued advances in environmental monitoring, genomic epidemiology, veterinary vaccination, and integrated surveillance have substantially improved outbreak prediction and response. Nevertheless, the increasing influence of climate change, globalization, and ecological transformation highlights the ongoing need for multidisciplinary collaboration. Strengthening partnerships among physicians, veterinarians, entomologists, ecologists, climatologists, public health authorities, and international organizations will remain essential for minimizing the future health and economic impacts of Rift Valley fever.

### 2.2. Crimean-Congo Hemorrhagic Fever Virus: A One Health Perspective

Crimean-Congo hemorrhagic fever virus (CCHFV) is among the most widely distributed tick-borne zoonotic viruses affecting humans and remains a significant global public health concern because of its extensive geographic range, high epidemic potential, and case-fatality rates that may approach 40% in severe outbreaks. As a member of the genus *Orthonairovirus* within the family *Nairoviridae*, CCHFV circulates through complex ecological systems involving ixodid ticks, domestic livestock, wildlife hosts, and humans [41]. These intricate transmission dynamics make CCHFV an archetypal One Health pathogen, highlighting the close interdependence of human, animal, and environmental health. Consequently, effective surveillance, prevention, and control require coordinated collaboration among physicians, veterinarians, entomologists, ecologists, environmental scientists, and public health authorities.

Crimean-Congo hemorrhagic fever (CCHF) was first recognized during outbreaks of severe hemorrhagic illness among Soviet military personnel stationed in Crimea during 1944–1945. More than two decades later, antigenic characterization demonstrated that the etiologic agent was identical to the Congo virus isolated in Central Africa, resulting in the unified designation Crimean-Congo hemorrhagic fever virus [42]. Since then, CCHFV has been documented in more than 50 countries across Africa, southeastern Europe, the Middle East, Central Asia, and western Asia, making it the most geographically widespread medically important tick-borne virus currently known [43,44]. Continued expansion into previously unaffected regions has intensified global concern regarding its epidemic potential. Its current geographic distribution is shown in Figure 2B.

The transmission ecology of CCHFV exemplifies the interconnected principles of the One Health framework. The virus is maintained primarily by hard ticks of the genus *Hyalomma*, which function as both vectors and natural reservoirs. Viral persistence within tick populations is facilitated through transstadial, transovarial, and venereal transmission, allowing infection to be maintained across multiple developmental stages and successive generations [41,44]. Numerous vertebrate hosts—including cattle, sheep, goats, camels, hares, hedgehogs, ostriches, and a variety of wild ungulates—develop transient viremia without overt clinical disease and serve as amplification hosts for feeding ticks [44]. Humans represent incidental hosts and generally do not contribute to sustained transmission, acquiring infection primarily through tick bites or direct contact with infected blood and tissues during livestock handling, slaughtering, veterinary procedures, or butchering [43].

Environmental conditions play a fundamental role in shaping the epidemiology of CCHFV. The abundance and geographic distribution of *Hyalomma* ticks are influenced by temperature, humidity, vegetation, host availability, and land-use practices. Increasing evidence indicates that climate change has facilitated the expansion of *Hyalomma* ticks into higher latitudes and elevations throughout Europe, thereby increasing the likelihood of CCHF emergence in regions previously considered unsuitable for sustained transmission [45,46]. Concurrently, agricultural intensification, habitat fragmentation, wildlife population dynamics, and increasing livestock trade have altered tick-host interactions and enhanced opportunities for viral dissemination [44]. These observations demonstrate that environmental change is a major determinant of CCHFV emergence and reinforce the importance of incorporating ecological monitoring into One Health surveillance systems.

Domestic livestock occupy a central position in the epidemiology of CCHF despite rarely exhibiting clinical illness. Following infection, cattle, sheep, goats, and camels develop short-lived viremia that is sufficient to infect feeding ticks and facilitate continued viral circulation within endemic ecosystems [41]. Consequently, individuals involved in livestock production—including farmers, veterinarians, abattoir workers, butchers, and animal traders—experience the greatest occupational risk of infection. Numerous outbreaks have been associated with the slaughter of viremic animals, particularly during periods of increased animal movement and religious festivals involving large-scale animal sacrifice [44]. These epidemiological patterns emphasize the importance of veterinary surveillance, livestock health monitoring, occupational education, and appropriate biosafety practices as key components of disease prevention.

Clinical manifestations of CCHF range from asymptomatic infection and mild febrile illness to severe viral hemorrhagic fever characterized by thrombocytopenia, hepatic injury, vascular dysfunction, disseminated intravascular coagulation, and multiorgan failure. Reported case-fatality rates generally range between 5% and 40%, depending upon viral strain, healthcare capacity, timeliness of diagnosis, and supportive clinical management [43]. Unlike many other tick-borne viruses, CCHFV also poses a significant risk for nosocomial transmission. Direct exposure to infected blood or bodily fluids has resulted in numerous hospital-associated outbreaks involving healthcare workers, emphasizing the necessity of strict infection prevention and control measures, rapid laboratory diagnosis, and appropriate biosafety protocols [41,47].

A major strength of the One Health approach lies in its capacity to detect viral circulation before human disease becomes widespread. Integrated surveillance systems that combine livestock serology, molecular testing of tick populations, wildlife surveillance, ecological risk assessment, and human disease reporting provide early warning of increased transmission risk [44]. Advances in geographic information systems (GIS), ecological niche modeling, and remote sensing have further enhanced the ability to identify regions favorable for *Hyalomma* tick establishment and predict seasonal periods of elevated transmission [45,46,48]. These multidisciplinary surveillance strategies allow public health authorities to implement targeted tick control, strengthen occupational protection, and increase community awareness before outbreaks occur.

Recent developments in molecular epidemiology have substantially improved understanding of CCHFV evolution and geographic spread. Whole-genome sequencing and phylogenetic analyses have demonstrated remarkable genetic diversity among viral lineages and have facilitated investigations into transboundary movement, viral reassortment, and regional patterns of emergence [44]. Increasing integration of genomic surveillance with veterinary, entomological, and epidemiological data has become an important component of contemporary One Health surveillance programs and enhances the capacity to monitor viral evolution while informing regional preparedness strategies.

International organizations have increasingly recognized CCHF as a priority zoonotic disease requiring coordinated multidisciplinary action. The World Health Organization, the Food and Agriculture Organization of the United Nations, and the World Organisation for Animal Health advocate integrated surveillance systems that combine human, animal, vector, and environmental health data to improve outbreak preparedness and response [11,43]. National One Health platforms established in several endemic countries now coordinate veterinary surveillance, laboratory diagnostics, vector monitoring, ecological investigations, and public health communication, demonstrating the practical application of multisectoral collaboration to reduce disease burden.

Although considerable progress has been made, significant challenges remain. No universally licensed human vaccine is currently available, although several recombinant, viral-vectored, DNA-based, and mRNA vaccine candidates are undergoing preclinical and clinical evaluation [44,49]. Likewise, sustainable tick control remains difficult because of the extensive geographic distribution of *Hyalomma* species, the diversity of wildlife hosts, and increasing acaricide resistance. Diagnostic capacity also remains limited in many endemic countries, resulting in delayed case recognition and underreporting. Continued investment in laboratory infrastructure, integrated surveillance systems, genomic monitoring, vector ecology, and interdisciplinary research will therefore be essential to improve preparedness and reduce future disease burden.

In summary, Crimean-Congo hemorrhagic fever virus represents one of the clearest examples of a zoonotic pathogen whose epidemiology is governed by complex interactions among vectors, wildlife, livestock, humans, and the environment. Climate change, expanding *Hyalomma* tick populations, international livestock movement, ecological transformation, and globalization continue to reshape the distribution and transmission dynamics of CCHFV. Addressing these challenges requires coordinated One Health strategies that integrate veterinary medicine, public health, ecology, entomology, environmental surveillance, and molecular epidemiology. As emerging zoonotic diseases continue to be influenced by anthropogenic environmental change, the One Health framework remains the most comprehensive approach for understanding, preventing, and mitigating the global threat posed by Crimean-Congo hemorrhagic fever virus.

### 2.3. Hantaviruses from a One Health Perspective: Integrating Human, Animal, and Environmental Health

Hantaviruses (HTVs) are globally distributed zoonotic pathogens whose emergence and persistence illustrate the close interdependence of human, animal, and environmental health. Classified within the family *Hantaviridae* (order *Elliovirales*), these viruses are maintained primarily in rodents, although an increasing number of genetically distinct HTVs have been identified in shrews, moles, and bats, expanding our understanding of their evolutionary diversity [50,51,52,53]. In contrast to most members of the class *Bunyaviricetes*, HTVs are transmitted directly from persistently infected mammalian reservoirs rather than through arthropod vectors. Human infections occur following accidental exposure to infected reservoir hosts and therefore represent classic zoonotic spillover events. Because viral transmission is influenced by wildlife ecology, environmental conditions, climate variability, land-use change, and human behavior, hantaviruses represent one of the most compelling examples of diseases that require a comprehensive One Health approach.

HTVs are traditionally divided into Old World and New World viruses according to their geographic distribution and the clinical syndromes they produce. Old World hantaviruses circulate throughout Europe and Asia and are primarily responsible for hemorrhagic fever with renal syndrome (HFRS), whereas New World hantaviruses are endemic throughout North and South America and cause hantavirus pulmonary syndrome (HPS), also referred to as hantavirus cardiopulmonary syndrome (HCPS) [50,51]. Although these syndromes differ clinically, both result from increased vascular permeability caused by virus-induced endothelial dysfunction coupled with an exaggerated host immune response rather than extensive viral cytopathology. Its current geographic distribution is shown in Figure 2C.

Among the Old World hantaviruses, Hantaan virus, Seoul virus, Dobrava-Belgrade virus, and Puumala virus are the principal causes of human disease. Hantaan virus, originally isolated in the Republic of Korea, is maintained by the striped field mouse (*Apodemus agrarius*) and causes severe forms of HFRS [54]. Puumala virus circulates in the bank vole (*Myodes glareolus*) and is responsible for nephropathia epidemica, a milder clinical form that predominates throughout northern and central Europe [51]. Seoul virus occupies a unique ecological niche because its primary reservoir, the Norway rat (*Rattus norvegicus*), is closely associated with human settlements and has achieved worldwide distribution through international commerce and urbanization [50]. Consequently, Seoul virus demonstrates how globalization, urban expansion, and human-mediated movement of reservoir hosts contribute to the worldwide dissemination of zoonotic pathogens.

The emergence of New World hantaviruses gained international attention following the 1993 outbreak of severe respiratory disease in the Four Corners region of the southwestern United States. Subsequent investigations identified Sin Nombre virus as the etiological agent and established the deer mouse (*Peromyscus maniculatus*) as its principal reservoir host [55]. Since then, several additional New World hantaviruses have been recognized, including Andes, Bayou, Black Creek Canal, Laguna Negra, and Choclo viruses, each associated with distinct rodent reservoirs and geographic distributions [50]. Among these viruses, Andes virus is particularly noteworthy because it remains the only hantavirus conclusively demonstrated to undergo efficient person-to-person transmission under natural conditions, although such events remain relatively uncommon and are largely restricted to South America [56].

The 2026 infectious-disease incident aboard the Dutch-flagged expedition cruise ship *M/V Hondius* has been a concrete example that rallied global authorities [57,58]. The outbreak was notified to the World Health Organization (WHO) on 2 May 2026 after several travellers developed severe acute respiratory illness, including fatal cases [57]. Epidemiological investigations suggested that the index infection was probably acquired before embarkation through exposure on land, rather than originating from the vessel itself. However, subsequent transmission was associated with travel aboard the *Hondius*, whose passengers and crew represented multiple countries. This international distribution required coordinated surveillance, contact tracing, quarantine measures and follow-up by national public-health authorities [57,58]. By 2 July 2026, the outbreak had resulted in 13 identified cases: 12 laboratory-confirmed Andes virus infections and one probable case. Three affected individuals died, corresponding to a reported case-fatality ratio of approximately 23% [59]. Symptomatic travellers were evacuated for medical care, while passengers and close contacts were repatriated, quarantined or actively monitored according to their estimated exposure risk [59,60]. Because Andes virus infection may have a prolonged incubation period, identified contacts were followed for 42 days after their last potential exposure. No further secondary cases were reported during this period. On 2 July 2026, WHO declared the outbreak contained and over [59]. Although the event was severe for the affected travellers, public-health authorities assessed the risk to the general population as low worldwide and very low within the European Union and European Economic Area, owing to the rarity of the virus, the apparent requirement for close-contact transmission, and the containment measures implemented [57,58]. This incident underscores the importance of analyzing a disease outbreak from a One Health perspective. As the initial victim was determined to have transmitted HTV to his spouse (i.e., the identification of a communicable disease), followed by the discovery that the initial victim visited an area in Argentina previously known to have been infested by rodents, the subsequent diagnosis of SNV (along with the events preceding the outbreak) strongly suggested that the virus was indeed transmitted from animal to human, ultimately causing a public health emergency.

The ecology of HTVs exemplifies the fundamental principles of the One Health framework. Each virus is closely adapted to a particular mammalian reservoir species, with which it has co-evolved over extended evolutionary timescales. Persistent infection develops in reservoir hosts without causing significant clinical disease, allowing infected animals to shed virus continuously in urine, saliva, and feces for prolonged periods [50]. Humans become infected primarily through inhalation of aerosolized virus-contaminated excreta, although transmission may also occur through direct contact with infected rodents or contaminated nesting materials [51]. The absence of arthropod vectors places particular emphasis on ecological interactions between wildlife reservoirs, environmental conditions, and human activities as the principal determinants of disease emergence.

Environmental variability strongly influences hantavirus transmission by regulating reservoir population dynamics and patterns of human exposure. Climatic conditions such as rainfall, temperature, and food availability affect rodent reproduction and survival, thereby altering the prevalence of infection within reservoir populations. One of the best-documented examples occurred during the 1993 Sin Nombre virus outbreak, when unusually heavy rainfall associated with the El Niño–Southern Oscillation increased primary productivity across the southwestern United States, leading to a marked expansion of deer mouse populations and subsequent spillover into humans [61]. Comparable ecological relationships have been described for Puumala virus in Europe, where mast years characterized by abundant seed production promote explosive increases in bank vole abundance that are followed by elevated incidences of HFRS [62].

Anthropogenic environmental change has become an increasingly important determinant of hantavirus emergence. Deforestation, agricultural expansion, mining, urbanization, and habitat fragmentation modify wildlife communities and frequently increase opportunities for contact between infected rodents and humans [63]. Expansion of agriculture and residential development into previously undisturbed ecosystems exposes forestry workers, farmers, military personnel, and rural communities to infected reservoir hosts. Likewise, deteriorating housing conditions, inadequate sanitation, and improper food storage encourage rodent infestations in both rural and urban environments. These factors are particularly important for Seoul virus, whose commensal rat reservoirs thrive in densely populated cities worldwide, illustrating how urban ecosystems can support the persistence of zoonotic pathogens [50].

The animal health component of the One Health framework is fundamental to understanding and preventing hantavirus transmission. Monitoring reservoir populations provides valuable information regarding viral circulation before human disease becomes apparent. Long-term surveillance of rodent abundance, reproductive activity, infection prevalence, and geographic distribution has become an important component of outbreak forecasting in several endemic regions [63]. Advances in molecular diagnostics and genomic sequencing have further facilitated the discovery of novel hantaviruses in rodents, shrews, moles, and bats, revealing that the diversity of the family *Hantaviridae* is considerably greater than previously recognized [52]. Integration of wildlife surveillance with molecular epidemiology has improved understanding of host adaptation, viral evolution, and mechanisms underlying zoonotic spillover.

The clinical manifestations of hantavirus infection differ considerably between Old World and New World viruses despite sharing similar pathogenic mechanisms. Hemorrhagic fever with renal syndrome is characterized by fever, thrombocytopenia, hypotension, acute kidney injury, and varying degrees of hemorrhage, with case-fatality rates ranging from less than 1% for Puumala virus infections to approximately 15% for infections caused by Hantaan or Dobrava-Belgrade viruses [51]. In contrast, hantavirus cardiopulmonary syndrome presents with rapidly progressive pulmonary edema, respiratory compromise, myocardial dysfunction, and cardiogenic shock, with reported mortality rates approaching 35–40%, particularly following infection with Sin Nombre or Andes viruses [50]. In both disease syndromes, vascular leakage results primarily from dysregulated immune responses targeting infected endothelial cells rather than direct destruction of host tissues.

Because no broadly licensed antiviral therapy is currently available and vaccines remain limited to a few countries using inactivated formulations against selected Old World hantaviruses, prevention relies primarily on reducing opportunities for human exposure to infected reservoir hosts [51]. Public health interventions therefore emphasize rodent-proofing of homes and workplaces, safe cleaning procedures that minimize aerosol generation, improved food storage, environmental sanitation, occupational education, and community awareness. These preventive measures are most effective when combined with integrated surveillance involving clinicians, veterinarians, wildlife biologists, ecologists, climatologists, and public health agencies that collectively identify environmental conditions associated with increased transmission risk [63].

Recent advances in One Health surveillance have increasingly incorporated genomic epidemiology, remote sensing, ecological niche modeling, and climate forecasting into hantavirus preparedness programs. International One Health initiatives coordinated by the World Health Organization, the Food and Agriculture Organization of the United Nations, the World Organisation for Animal Health, and the United Nations Environment Programme have further emphasized the importance of integrating wildlife surveillance, environmental monitoring, and human health surveillance to strengthen preparedness for emerging zoonotic diseases [11].

Climate change is expected to further reshape the epidemiology of hantaviruses by altering the geographic distribution, seasonal abundance, and reproductive dynamics of reservoir hosts. Changes in temperature, precipitation, vegetation productivity, and biodiversity are likely to influence both rodent population dynamics and opportunities for human exposure, potentially facilitating the emergence of hantaviruses in regions where transmission has historically been uncommon [63]. Consequently, predictive ecological models that combine climate data, remote sensing technologies, wildlife surveillance, and epidemiological information are becoming increasingly valuable components of One Health-based early warning systems.

In conclusion, both Old World and New World hantaviruses exemplify the importance of the One Health framework for understanding and mitigating emerging zoonotic diseases. Their transmission depends upon complex interactions among wildlife reservoirs, environmental conditions, climate variability, and human activities. Ongoing environmental change, urbanization, globalization, and habitat disturbance continue to influence rodent ecology and increase opportunities for zoonotic spillover. Effective prevention therefore requires sustained interdisciplinary collaboration integrating wildlife ecology, veterinary medicine, environmental science, molecular epidemiology, public health, and clinical medicine. As global environmental pressures continue to intensify, the One Health approach will remain essential for predicting disease emergence, strengthening surveillance, and reducing the worldwide impact of hantavirus infections.

## 3. Concluding Remarks

While the One Health concept can be applied to a wide variety of pathogens, bunyaviruses represent a near prototypical public health threat which requires such an approach. Due to their (largely) zoonotic transmission cycles, as well as their well-studied interactions between humans, animals and their surrounding environments, a better understanding of these three interacting factors has already been shown to be effective in predicting and reducing their threat to public health. Recent advances in molecular diagnostics, pathogen genomics, geographic information systems, ecological niche modeling, remote sensing, and climate forecasting have transformed the ability to detect, monitor, and predict zoonotic disease emergence. When integrated within One Health surveillance systems, these technologies facilitate the early identification of high-risk areas, improve outbreak forecasting, and support timely implementation of targeted interventions before widespread transmission occurs. Equally important, coordinated surveillance programs that combine human, animal, vector, and environmental data have strengthened the capacity to recognize changes in pathogen circulation and rapidly respond to emerging threats.

Apart from all being of the same phylogenetic class, RVFV, CCHFV and HTV are of particular importance from a One Health perspective, as all three viruses are vector-transmitted, highly pathogenic, and have demonstrated a geographic range covering multiple continents. With population centres increasing and climate change expanding the range of transmission vectors, one can reasonably expect that all three pathogens will continue to be a threat to public health. Ultimately, the One Health framework provides not only a conceptual foundation for understanding the ecological complexity of zoonotic diseases but also a practical roadmap for mitigating their impact. As environmental and societal pressures continue to evolve, sustained interdisciplinary collaboration and international cooperation will be indispensable for improving global health security and reducing the burden of emerging and re-emerging viral diseases. A summary comparing and contrasting the One Health approach currently used to address the public health threats of RVFV, CCHFV and HTV is summarized in Table 1.

As previously mentioned, the One Health approach underscores the importance of understanding that humans, animals and the environment all play essential roles in maintaining public health. Although widely understood to be zoonotic, one of the unsolved questions of the COVID-19 pandemic involves the precise mode of transmission (i.e., the initial host of the virus), and whether or not any intermediate hosts were involved. Furthermore, were the interactions between veterinary, public health and environmental authorities (on a regional, national and international level) sufficient? While the WHO/FAO/UNEP/WOAH quadripartite alliance promises to address these deficiencies, their prospects in establishing an effective One Health strategy will still be limited by how government agencies fund, implement and cooperate both internally and internationally.

## Figures and Tables

**Table 1:** Comparative One Health characteristics of RVFV, CCHFV, and HTV.

Feature	Rift Valley Fever Virus (RVFV)	Crimean-Congo Hemorrhagic Fever Virus (CCHFV)	Hantaviruses (HTV)
**Main transmission system**	Mosquito–livestock–human cycle, with mosquitoes acting as vectors and livestock as important amplification hosts.	Hyalomma tick–livestock/wildlife–human cycle.	Wildlife reservoir–environment–human transmission, primarily involving rodents.
**Key animal hosts**	Sheep, cattle, goats, camels, and other ruminants; wildlife may contribute to maintenance.	Cattle, sheep, goats, camels, hares, and wild ungulates.	Mainly rodents, although shrews, moles, and bats can harbor hantaviruses.
**Environmental drivers**	Rainfall, flooding, temperature, standing water, and mosquito abundance.	Temperature, humidity, vegetation, land use, and conditions affecting Hyalomma distribution.	Rainfall, temperature, vegetation, food availability, habitat change, and rodent population dynamics.
**Climate-change concern**	Climate variability may increase mosquito populations and create conditions favorable for outbreaks.	Warmer conditions may expand Hyalomma populations into previously unsuitable regions.	Climate variability can alter rodent abundance and distribution, modifying human spillover risk.
**Major human Exposure**	Contact with infected livestock, animal tissues, mosquitoes, and contaminated biological materials.	Tick bites and contact with infected blood or tissues from livestock; healthcare-associated transmission can occur.	Inhalation or contact with virus-contaminated rodent urine, feces, saliva, or nesting materials.
**Priority surveillance**	Livestock disease and abortion monitoring, mosquito surveillance, climate monitoring, and human cases.	Tick and livestock surveillance, wildlife monitoring, ecological modeling, and human disease surveillance.	Rodent abundance and infection monitoring combined with environmental and human disease surveillance.
**Principal prevention**	-Livestock vaccination, mosquito control, animal-movement management, and occupational protection.	-Tick control, livestock surveillance, occupational protection, infection-control measures, and rapid diagnosis.	-Rodent control, rodent-proofing, environmental sanitation, safe cleaning, and public education.
**One Health focus**	-Integrating livestock, mosquitoes, climate, and human health to anticipate outbreaks.	-Linking ticks, wildlife, livestock, environmental change, and human health.	-Connecting wildlife reservoirs, environmental conditions, human behavior, and spillover risk.
**Vaccines**	-Inactivated vaccines available for animals, -No licensed vaccines for humans; experimental vaccines available for veterinary and laboratory personnel	-No licensed vaccine available	-No licensed vaccine in North America or Europe -Inactivated vaccines in Asia against HFRS
**Diagnostics**	-Reverse Transcriptase Polymerase Chain Reaction (RT-PCR) -IgG and IgM antibody enzyme-linked immunosorbent assay (ELISA) -Virus isolation by cell culture	-Reverse Transcriptase Polymerase Chain Reaction (RT-PCR) -Serum neutralization -IgG and IgM antibody enzyme-linked immunosorbent assay (ELISA) -Virus isolation by cell culture	- Patient history (i.e., rodent exposure) -Reverse Transcriptase Polymerase Chain Reaction (RT-PCR) -IgG and IgM antibody enzyme-linked immunosorbent assay (ELISA)
**International Organizations Involved**	-World Health Organization -Food and Agricultural Organization (FAO) -World Organization of Animal Health (WOAH)	World Health Organization	World Health Organization

## Data Availability

No new data was created. Data sharing is not applicable to this article.
